# The Role of Prevention and Early Detection in Skin Tumors: Correlation Between Educational Level and Tumor Stage at Diagnosis

**DOI:** 10.3390/jcm14207321

**Published:** 2025-10-16

**Authors:** Delia Nicoara, Ioan Constantin Pop, Maximilian Vlad Muntean, Radu Alexandru Ilies, Robert Nan, Patriciu Andrei Achimas-Cadariu

**Affiliations:** 1 Faculty of Medicine, “Iuliu Hațieganu” University of Medicine and Pharmacy, 400012 Cluj-Napoca, Romania; drdelianicoara@gmail.com (D.N.); ilies.radu.alexandru@elearn.umfcluj.ro (R.A.I.); 2 Department of Quality Management, “Prof. Dr. I. Chiricuță” Institute of Oncology, 400015 Cluj-Napoca, Romania; 3 Department of Plastic Surgery, “Prof. Dr. I. Chiricuță” Institute of Oncology, 400015 Cluj-Napoca, Romania; nanrobert7@gmail.com; 4 Department of Plastic and Reconstructive Surgery, “Iuliu Hațieganu” University of Medicine and Pharmacy, 400012 Cluj-Napoca, Romania; 5 Department of Surgical Oncology and Gynecologic Oncology, “Iuliu Hațieganu” University of Medicine and Pharmacy, 400012 Cluj-Napoca, Romania; pachimas@umfcluj.ro; 6 Department of Surgical Oncology, “Prof. Dr. I. Chiricuță” Institute of Oncology, 400015 Cluj-Napoca, Romania

**Keywords:** skin cancer, melanoma, education, diagnostic delay, health literacy, prevention

## Abstract

**Background/Objectives**: Representing the most common malignancy worldwide, skin cancer requires timely detection to improve prognosis. Both educational level of the patients and health literacy are important variables in terms of prevention and diagnosis in early stages of the disease, but data from Central and Eastern Europe are limited. **Methods**: We realized a prospective observational study that included 76 patients who were diagnosed with skin cancer and treated at the “Prof. Dr. I. Chiricuță” Institute of Oncology in Cluj-Napoca, Romania. Demographic, clinical, histopathological, and psychosocial data were collected in a standardized form. The primary aim was the measurement of diagnostic delay, defined as the interval since symptom onset until diagnosis. Secondary variables included education level, place of residence, participation in awareness campaigns and understanding capacity. Statistical analyses were applied. **Results**: The mean age in the cohort was 58.3 years; 52.6% were male and 84.2% were urban residents. The most frequent histological type was melanoma (47.4%), followed by basal cell carcinoma (36.8%), and squamous cell carcinoma (10.5%). The median delay in diagnostic was equal to 3 weeks. Education level was significantly related to earlier presentation (Kruskal–Wallis, *p* = 0.043), with shorter delays noticed in patients with university or postgraduate degrees (compared to those with secondary education). However, there were no significant differences between patients with rural and urban provenience (*p* = 0.483). Patients’ capacity of understanding showed no correlation with diagnostic delay, but their prior participation in awareness campaigns was strongly associated with higher comprehension (*p* < 0.001). Also, skin self-examination did not significantly impact time to diagnosis (*p* = 0.86). **Conclusions**: Higher levels of education and patients’ exposure to awareness campaigns might represent predictors of shorter diagnostic delay, highlighting the impact of public health initiatives and targeted educational strategies to improve early detection of skin cancers in Romania. However, the findings must be interpreted in light of the study’s limitations, namely the relatively small sample size and single-center design.

## 1. Introduction

Nowadays, skin cancer represents the most prevalent malignancy in the United States, with an incidence which continues to rise significantly. Current estimates suggest that one in six individuals will develop some form of skin cancer over the course of their lifetime. Clinically, cutaneous malignancies are categorized into two major groups: melanoma and nonmelanoma skin cancers (NMSCs). The latter category is predominantly composed of basal cell carcinomas (BCC) and squamous cell carcinomas (SCC), accounting for an estimated 1.2 million new cases annually in the U.S [1].

While BCC and SCC are rarely associated with mortality, they are characterized by rapid local progression and, if left untreated, can lead to considerable tissue destruction and functional impairment. Conversely, melanoma comprises approximately 5% of all skin cancer diagnoses, with a disproportionately higher mortality rate, responsible for roughly 15% of skin cancer-related deaths. Even though it is more frequently diagnosed in older individuals, melanoma remains the most common malignancy among women aged 25–29 and the second most common in those aged 30–34. Tumor thickness (Breslow depth) remains the most significant prognostic factor in melanoma, underscoring the importance of early detection and timely intervention to optimize clinical outcomes [1].

Beyond melanoma and the keratinocyte-derived NMSCs, a diverse array of less common cutaneous malignancies exists, though these are met less frequently in routine clinical practice. Traditionally, cutaneous neoplasms are classified according to their tissue of origin—arising from the epidermis, dermis, adnexal structures, or through secondary involvement from systemic malignancies [1].

Beyond epidemiology, cutaneous SCC exhibits marked biological heterogeneity and one of the highest mutational burdens among solid tumors. Recent profiling studies in head-and-neck SCC emphasize recurrent UV-signature mutations (C > T transitions) involving key tumor suppressor genes such as TP53 and CDKN2A, along with alterations in NOTCH1/2 and HRAS, converging on p53, cell-cycle, RTK/RAS and PI3K signaling pathways. Interestingly, higher overall mutation counts have been associated with increased tumor immunogenicity, supporting a role for immune surveillance in clinical behavior [2]. These data highlight that, alongside social determinants (education, awareness), intrinsic tumor biology contributes substantially to outcomes in skin cancer.

Squamous cell carcinoma (SCC) represents the second most common type of skin cancer in the U.S. but is responsible for a higher mortality than basal cell carcinoma. It accounts for approximately 20% of skin cancers, with over one million new skin cancer cases estimated in 2008. SCC often predominantly affects elderly, fair-skinned individuals and arises mainly due to chronic ultraviolet (UV) exposure (especially UVB radiation). Around 80% of cases occur on sun-exposed skin. The pathogenesis of SCC is multifactorial, involving both genetic susceptibility and environmental factors. DNA damage to keratinocytes from UV radiation is considered a key carcinogenic driver. Additional risk factors include long-term immunosuppression (e.g., in organ transplant recipients), with incidence rates rising significantly over time post-transplant. Immunosuppressive regimens contribute by promoting tumorigenic cytokines. Human papillomavirus (HPV), particularly types 16 and 18, is involved in the development of genital and head and neck SCC, affecting both immunocompetent and immunosuppressed individuals. Chronic inflammatory dermatoses (such as venous ulcers, discoid lupus erythematosus, erosive lichen planus, and lymphedema) can also undergo malignant transformation into SCC. Clinical changes in chronic skin lesions, particularly ulceration or induration, require biopsy to rule out neoplastic progression [1].

Basal cell carcinoma (BCC) is the most common malignancy in humans, accounting for approximately 75% of all skin cancers. First described in 1827 by Arthur Jacob, BCC is named for its histologic resemblance to the basal cells of the epidermis. The incidence continues to rise, with over one million new cases estimated in the U.S. by 2008. BCC predominantly affects fair-skinned individuals, though it can also occur in those with darker skin types. Intermittent UV exposure during childhood (rather than continuous exposure in adulthood) is considered the primary etiologic factor. UV wavelengths around 293–380 nm are particularly implicated. There is also an inverse relationship between geographic latitude and BCC incidence, and potential associations have been identified with vitamin D receptor polymorphisms. As with squamous cell carcinoma, immunosuppressed patients (especially those who undergo solid organ transplantation) are at increased risk. Most BCCs occur on sun-exposed areas, particularly the head and neck, though lesions may also appear on protected sites. Clinically, about 60% of BCCs present as nodulo-ulcerative lesions, also known as “rodent ulcers”, beginning as translucent nodules with surface telangiectasias and progressing to ulceration with rolled borders. While BCCs rarely metastasize, they can be locally invasive and, if neglected, may cause significant tissue destruction, including rare cases of intracranial or bony invasion [1].

On the other hand, melanoma is a highly aggressive skin cancer originating from melanocytes, found in the skin and other pigmented tissues. Although it accounts for a lower per cent of skin malignancies, it is the deadliest form. It primarily affects Caucasians of both sexes and has a poor prognosis once metastatic. Early detection is critical, and according to ESMO guidelines, accurate staging and, in some cases, mutation testing are essential for optimal management [3].

In the past decade, melanoma mortality rates have decreased by an average of 2.9% annually, despite a 1.5% yearly increase in new cases. This decline is largely attributed to improved early detection. However, significant problems remain, as there are estimated 6850 melanoma-related deaths which occurred in the U.S. in 2020, that may have been preventable with earlier diagnosis [4,5,6,7].

Malignant melanoma is classified into several subtypes based on clinicopathological features. The most common form is superficial spreading melanoma, followed by lentigo maligna (LM) melanoma, nodular melanoma, acral lentiginous melanoma, desmoplastic melanoma, and other rarer variants. LM and superficial spreading melanoma are in situ lesions confined to the epidermis and adnexal epithelium, which are generally associated with a better prognosis. In contrast, variants with dermal invasion have a worse prognosis. While most melanomas are pigmented, amelanotic melanomas (less than 2%) are particularly challenging to diagnose clinically due to their lack of pigmentation [1,8].

Treatment options for melanoma depend on various tumor characteristics such as location, stage, and genetic profile. These may include surgical excision, chemotherapy, radiotherapy, photodynamic therapy (PDT), immunotherapy, or targeted therapy [9,10]. For stage I–IIIB melanoma, surgery remains the cornerstone of treatment, with adjuvant targeted or immunotherapy recommended to enhance survival outcomes [3,10].

According to SEER data from 2011 to 2017, the overall 5-year survival rate for melanoma has improved to 93.3%, up from 81.9% in 1975. Survival varies significantly by stage at diagnosis: 99.4% for stage I–II, 68.0% for stage III, and 29.8% for stage IV. Moreover, 83% of melanomas are diagnosed at an early stage (I–II), while only 4% are identified at stage IV [11].

Public health initiatives in countries like Australia, particularly the SunSmart program, have demonstrated a significant success in reducing melanoma incidence through sustained education campaigns, school-based policies, and promotion of sun-protective behaviors such as wearing hats, seeking shade, and using sunscreen [12]. Despite strong evidence from randomized controlled trials proving that regular sunscreen use can reduce melanoma risk decades later, adherence in the U.S. remains suboptimal, as less than 40% of individuals consistently use sunscreen on sun-exposed areas. Programs like SunWise (in the U.S.) have shown promising results, preventing thousands of skin cancer cases and deaths with a favorable cost–benefit ratio [13]. These findings highlight the need for increased investment in comprehensive sun safety education, policy development, and community engagement to strengthen primary prevention efforts and decrease the incidence of melanoma and other skin cancers.

In parallel with prevention and awareness strategies, technological innovations are rapidly emerging to support early detection. In recent years, novel technologies have emerged to enhance skin cancer detection. Hyperspectral imaging, combined with machine learning algorithms, has demonstrated improved overall accuracy in distinguishing malignant from benign cutaneous lesions, particularly in challenging scenarios such as differentiating between actinic keratosis, basal cell carcinoma or seborrheic keratosis. While these tools represent an important step toward precision diagnostics, their availability remains limited, and their implementation is resource-dependent. Therefore, in many Central and Eastern European settings, timely patient presentation (driven by education and awareness) continues to play a decisive role in achieving early diagnosis [14].

The aim of this study is to evaluate the relationship between the educational level of the patients and the stage of skin cancer at diagnosis, to make it possible to determine if limited health awareness contributes or not to diagnostic delay. The study also has the objective to describe the demographic and clinical features of patients, to explore potential urban–rural differences regarding access to timely diagnosis (and treatment), and to assess the influence of several characteristics like the patients’ level of understanding and their prior exposure to awareness campaigns. Also, we aim to examine the role of prevention behaviors such as skin self-examination related to diagnostic delay.

## 2. Materials and Methods

### 2.1. Study Design, Population, Inclusion and Exclusion Criteria

This study was conducted as a prospective observational study aimed at evaluating patients diagnosed with skin cancer and treated at the Oncology Institute. The Ethics Committee of the “Prof. Dr. Ion Chiricuță” Oncology Institute, Cluj-Napoca, decided that this study (Assessment Report No. 347/12 August 2025, Application No. 7500/11 August 2025) did not require prior ethical approval, because it was limited to the analysis of anonymized data extracted from routine medical practice. The Committee confirmed that the study was realized in accordance with the principles of the Declaration of Helsinki, as all patients provided written informed consent for the use of anonymized data for scientific purposes.

The study population included patients diagnosed with skin cancer who received treatment at the “Prof. Dr. I. Chiricuță” Institute of Oncology (IOCN), Cluj-Napoca, Romania. Participants were enrolled consecutively over the study period, following informed consent.

Inclusion Criteria:

- Patients aged 18 years or older
- Histologically confirmed diagnosis of skin cancer
- Undergoing treatment at IOCN (Cluj-Napoca, Romania)
- Provided informed consent for participation.

Exclusion Criteria:

- Patients with recurrent or metastatic disease at presentation
- Patients aged under 18 years old
- Incomplete medical records or inability to obtain informed consent.

Recurrent or metastatic cases were excluded because the study aimed to evaluate diagnostic delay exclusively in first-time diagnoses. Inclusion of relapsed patients would have introduced bias related to prior treatment history, surveillance practices, and disease biology, which could negatively influence the analysis.

In Romania, patients with suspected skin cancer typically follow a referral pathway starting with the initial consultation in primary care or dermatology offices. When a suspicious lesion is identified, the patient receives a referral letter to a specialized center. Cases requiring surgical excision are directed to departments of plastic surgery or dermatology, with histopathological confirmation following excision. This referral system explains why the patients included in our study were admitted to the Department of Plastic Surgery at IOCN with a medical letter, ensuring that cases centralized in tertiary centers are carefully evaluated and treated.

### 2.2. Data Collection and Statistical Analysis

Clinical, demographic, and pathological data were collected prospectively for all eligible patients. The data collection form included information such as:

- Age, sex, and education level
- Tumor location, histologic type, and stage at diagnosis
- Type of treatment received
- Time from symptom onset to diagnosis
- Risk factors (e.g., sun exposure, immunosuppression, family history)
- Patient’s Understanding Level (assessed by physician)

Data Collection Form:

1. Demographic Data
  - Patient code (anonymized): __________
  - Age: ______ years
  - Sex: □ Male ☐ Female
  - Place of residence: ______________________
  - Area of origin: ☐ Urban ☐ Rural
  - Education level:
    ☐ No formal education
    ☐ Primary school (grades 1–4)
    ☐ Middle school (grades 5–8)
    ☐ High school
    ☐ Post-secondary/vocational training
    ☐ University degree
    ☐ Postgraduate studies
2. Clinical Data
  - Date of symptom onset (month/year): ____________
  - Date of first medical consultation: ____________
  - Time from symptom onset to diagnosis: ________ weeks/months
  - Tumor location: _______________________
  - Tumor size at presentation: ________ mm
  - Clinical signs:
    ☐ Ulceration
    ☐ Bleeding
    ☐ Pain
    ☐ Itching
    ☐ Rapid growth
3. Histopathological Data
  - Histologic type:
    ☐ Basal cell carcinoma (BCC)
    ☐ Squamous cell carcinoma (SCC)
    ☐ Melanoma
    ☐ Other (specify): ___________________
  - Invasion depth/TNM stage (if applicable): ____________________
  - Resection margins: ☐ Clear ☐ Involved
4. Risk Factors
  - Chronic sun exposure: ☐ Yes ☐ No
  - Use of sun protection: ☐ Yes ☐ No
  - Family history of skin cancer: ☐ Yes ☐ No
  - Immunosuppressive conditions: ☐ Yes ☐ No
  - Occupation (UV exposure): ___________________
5. Treatment Administered
  - Type of treatment:
    ☐ Surgical excision
    ☐ Excision + local reconstruction
    ☐ Radiotherapy
    ☐ Other methods (specify): _____________________
  - Complete treatment: ☐ Yes ☐ No
  - Postoperative complications: ☐ Yes ☐ No (if yes, specify): ________________
6. Other Relevant Observations:
7. Patient’s Understanding Level (assessed by physician)

Rate the perceived level of the patient’s understanding of their medical condition, on a scale from 1 to 5:

☐ 1—Not at all (does not understand the medical issue)
☐ 2—Understands very little (limited understanding, often confused)
☐ 3—Moderate understanding, but lacks awareness of the severity
☐ 4—Good understanding, aware of the seriousness of the condition
☐ 5—Full understanding, actively involved in decision-making and treatment.

The questionnaire was completed by patients during their first consultation in the Department of Plastic Surgery, after being referred with a medical letter from their general practitioner or dermatologist. Before filling in the form, patients were informed about its purpose and assured that the data would be anonymized. The questionnaire was self-administered in written format, but the attending physician was present throughout the process to provide clarification for unclear items, to ensure that all questions were answered, and to minimize missing data. After completion, each questionnaire was reviewed together with the patient by the physician, in order to verify the consistency of answers and to reduce the risk of recall bias or misinterpretation. This approach allowed us to obtain accurate and complete information on demographic, clinical, and psychosocial variables.

By collecting and analyzing these variables, we aim to identify potential associations between the patient’s educational level and the stage of disease at the time of diagnosis. This approach seeks to determine whether a lower level of education is linked to delayed medical consultation and more advanced tumor stages. Gaining insight into this relationship could inform targeted public health interventions, enhance early detection efforts, and guide educational initiatives for populations with limited health awareness or restricted access to medical information.

The statistical analysis was performed using both descriptive and inferential methods, allowing for a comprehensive overview of the data and also to identify significant associations and patterns. Descriptive statistics were used to summarize and characterize the main features of our dataset, while inferential techniques were applied to test hypotheses between groups and draw conclusions beyond the cohort sample. All these analyses were performed with the support of SPSS v26.0, which facilitated accurate data processing and interpretation. Microsoft Word was used for construction of the database and further processing of the raw data. A *p*-value < 0.05 was considered to be statistically significant.

## 3. Results

### 3.1. Patient Characteristics

In total, 76 patients who had been diagnosed with skin cancer were analyzed. Their age varied between 26 and 83 years; the average value was 58.3 years, the median equal to 60, SD was 14.5 and IQR 50–64. Our cohort included 40 males (52.6%) and 36 females (47.4%). Most patients were residents of urban areas (n = 64; 84.2%), while 12 patients (15.8%) originated from rural settings. Educational backgrounds were the following: university 28 (36.8%), postgraduate 24 (31.6%), post-secondary/vocational 12 (15.8%), and high school 12 (15.8%) (as represented in Table 1).

### 3.2. Clinical and Histopathological Data

The most frequently reported symptom at presentation was rapid growth, noticed in 56 patients (73.7%). Bleeding was met in 20 patients (26.3%), while patients complained of pain in 8 cases (10.5%). As shown in Table 2, other less common manifestations met in the cohort were ulceration and itching, each of them encountered in 4 patients (5.3%).

**Figure 1:**
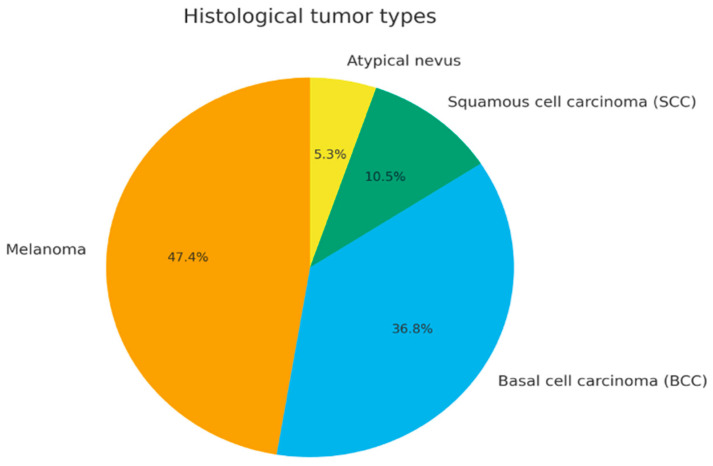
Distribution of histological tumor types (pie chart).

According to histopathological reports, melanoma represented the most common diagnosis (47.4%), followed by basal cell carcinoma (36.8%), squamous cell carcinoma (10.5%), and atypical nevus (5.3%) (Figure 1). Overall, the median interval from symptom onset to diagnosis was equal to 3 weeks (average value of 3.6 weeks).

When stratified by residence, melanoma accounted for 48.4% of urban cases vs. 41.7% of rural cases, BCC for 35.9% vs. 41.7%, and SCC for 10.9% vs. 8.3%, respectively. These differences were not statistically significant (chi-square test, *p* = 0.74).

### 3.3. Diagnostic Delay and Educational Level

**Figure 2:**
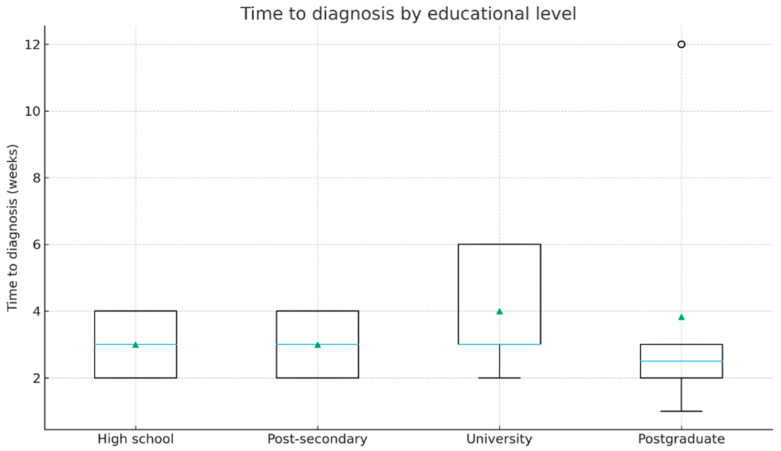
Time from symptom onset to diagnosis according to educational level (boxplot). The horizontal blue line represents the median. The green triangles indicate the mean values.

Patients with higher educational level had a tendency to present earlier compared to those with lower educational level. Group medians were the following: high school 3.0 weeks (IQR 2.0–4.0), post-secondary 3.0 (2.0–4.0), university 3.0 (3.0–6.0), postgraduate 2.5 (2.0–3.0). Notably, the differences between the education groups were statistically significant (Kruskal–Wallis, *p* = 0.043). Their distribution is illustrated in Figure 2.

### 3.4. Urban–Rural Differences in Diagnostic Delay

**Figure 3:**
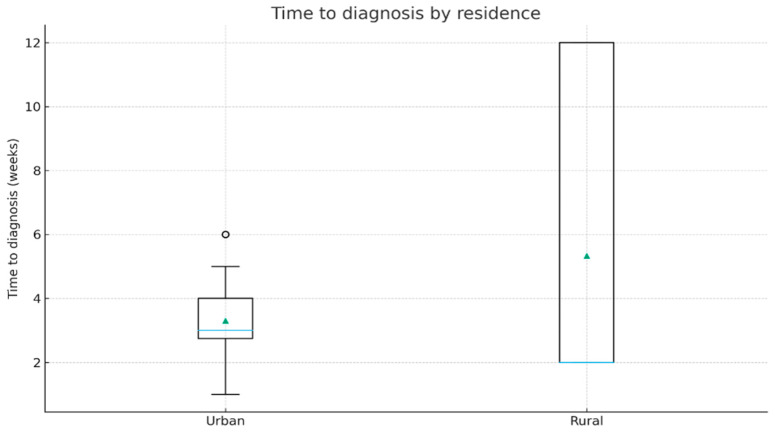
Time to diagnosis by residence (boxplot). The horizontal blue line represents the median; green triangles indicate the mean values.

Median delay until diagnostic was 3.0 weeks (IQR 2.75–4.0) in urban patients and 2.0 weeks (IQR 2.0–12.0) in rural patients. Even though rural patients presented a wider dispersion (long upper tail), the difference in distributions was not statistically significant (Mann–Whitney U, *p* = 0.483). Figure 3 provides further details.

### 3.5. Patient Understanding and Access to Information

**Figure 4:**
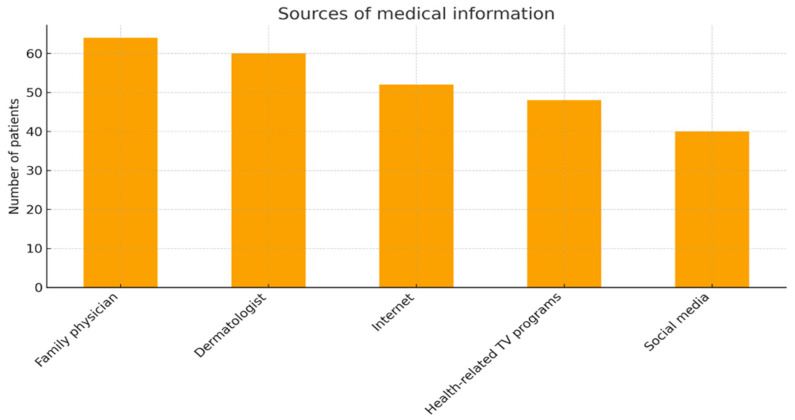
Sources of medical information (bar chart).

The understanding capacity of patients was assessed by physicians and ranged from 1 to 5, in most of the cases scoring 3–4 (Table 3). The correlation between the evaluated understanding capacity and the duration until diagnostic was weak and did not present statistical significance (Spearman’s rho = −0.17, *p* = 0.15). Out of the 76 patients, 16 (21.1%) reported prior participation in awareness campaigns on skin cancer, 56 (73.7%) had not participated, and 4 (5.3%) could not recall participating. Regarding sources of medical information, the most frequently mentioned were family doctors (84.2%) and dermatologists (78.9%), followed by the internet (68.4%), social media (52.6%), and TV/radio programs (52.6%). Patients who participated in awareness campaigns had significantly higher understanding levels compared to those who did not (chi-square test, *p* < 0.001). The patients’ reported sources of medical information are emphasized in Figure 4.

### 3.6. Self-Examination and Diagnostic Delay

**Figure 5:**
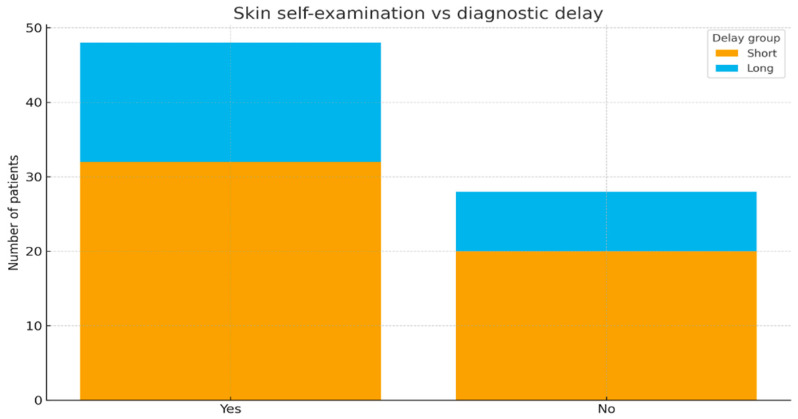
Skin self-examination versus diagnostic delay (stacked bar).

No significant association was found between the practice of skin self-examination and shorter time intervals until diagnostic (chi-square test, *p* = 0.86). The distribution of long versus short delays (depending on self-examination status) is displayed in Figure 5.

### 3.7. Summary of Inferential Statistics

All statistical tests that were performed, accompanied by their outcomes (*p*-values), and interpretations are summarized below, in Table 4.

### 3.8. Detailed Descriptive Statistics for Key Comparisons

In order to facilitate transparency and replication, we display in Table 5 and Table 6 the medians and IQRs by education and by residence, respectively.

## 4. Discussion

### 4.1. Interpretation of the Main Findings

Several key findings derive from our results. First, the demographic features of the cohort suggest a predominance of middle-aged and older adults, with an average value of age equal to 58 years. The nearly equal distribution of sexes, combined with the higher proportion of urban residents, indicate that access to specialized care in oncology is more commonly linked to urban populations, even if the incidence of skin cancer is often higher in rural areas due to increased exposure to risk factors. The educational level in our cohort was relatively high, with more than two-thirds of patients presenting university or postgraduate degrees.

Second, according to histopathology, melanoma was dominant in almost half of all cases, confirming that this disease presents an increasing incidence in Central and Eastern Europe, while basal cell carcinoma was present in more than one-third of all cases. This distribution (even if it may be counter-intuitive) reflects both the aggressive behavior of melanoma and the potential bias of referral toward a tertiary oncology center, where more complex cases are centralized.

Third, the delay until diagnosis was, overall, short (median value of 3 weeks), but education constituted a significant variable for predicting time to presentation. Patients with higher educational levels (university or postgraduate) were diagnosed earlier, while those with only secondary education tended to experience delays which were longer. The statistically significant association between educational level and diagnostic interval highlights the important role of health awareness in influencing prevention behaviors and timely diagnosis.

It is also important to note that the diagnostic interval we noticed (median of 3 weeks) is shorter than initially anticipated. This discrepancy might be justified by the referral pathway in Romania, where suspected cancer cases are centralized to tertiary oncology centers, facilitating faster confirmation of diagnosis once patients reach specialized care. Furthermore, our cohort included predominantly urban and highly educated individuals, who are more likely to seek medical attention earlier.

On the other hand, the difference between patients originating from rural and urban areas (although it is suggestive of a larger variability in rural areas) did not present statistical significance. This might be caused by the relatively small proportion of patients originating from rural areas in our cohort, but it also indicates that education could have a higher impact compared to geographic residence.

In addition, the assessment of clinical signs showed that rapid tumoral growth was the most common manifestation, followed by bleeding and itching, while ulceration and pain were relatively rare. All these findings align with the natural history of skin cancers (particularly melanoma, that usually presents as a rapidly enlarging pigmented lesion).

Moreover, the patients’ level of understanding the disease was globally moderate to good, with physician-assessed scores varying around 3–4 out of 5 on the scale. Although the correlation between understanding capacity and diagnostic delay did not reach the level of statistical significance, patients who had been previously exposed to awareness campaigns demonstrated significantly better comprehension. This illustrates the efficacy and benefits of targeted educational interventions and underscores the vital role of public health initiatives in raising awareness of skin cancer.

Preventive behaviors (like skin self-examination) did not show a significant association with earlier diagnosis. This might be explained by the fact that the majority of patients presented only when major and visible changes like rapid growth or bleeding occurred, emphasizing that self-examination alone is not sufficient in the absence of medical follow-up and health literacy.

Altogether, our findings indicate that educational level and continuous exposure to awareness campaigns are stronger predictors of early presentation to specialists compared to either residence or self-reported preventive practices.

### 4.2. Overview of the Existing Literature

Lacson et al. (2023) [15] performed a study in which they included Hispanic adults in Florida and Puerto Rico with the aim to evaluate behaviors of skin cancer prevention and other related psychosocial factors. Low levels of sun-protective practices were found, with nearly one-third reporting sunburn in the last year, despite increased reported self-efficacy and belief in preventive measures. Some differences appeared depending on language and location: English-speaking participants located in Tampa reported more behaviors at risk, while the Spanish-speaking Puerto Ricans showed a greater concern and also perceived risk. The authors conclude that interventions adapted to culture and linguistics, guided by Protection Motivation Theory (PMT), can enhance prevention efficacy in some populations [15].

Wu et al. (2022) [16] conducted a randomized trial in which young adults were included, with the objective to assess the impact of education in skin cancer prevention, personalized UV photography, and MC1R genetic testing (alone or in combination). Although all these interventions improved prevention behaviors, the combination of UV photography with genetic testing approach was responsible for the most consistent positive effects on sun protection and reduced risky behaviors. Their conclusion was that the integration of multiple risk communication strategies might enhance prevention of skin cancer in young adults [16].

In their study, Maleki et al. (2022) [17] tested an educational intervention (based on PMT) among 104 male students (with an average age of 13 years) in Isfahan, Iran. After five sessions, the behavior score of the intervention group increased from 39.6 (SD 21.4) to 74.7 (SD 23.5) (*p* < 0.001), while in the control group, no significant change was reported. All PMT characteristics improved significantly (*p* < 0.001), except for response cost. The authors concluded that short-term PMT-based programs can efficiently improve sun-protective behaviors of adolescents [17].

Parsons et al. (2021) [18] performed a study in which they followed 97 child–parent groups wearing UVR monitors for a period of 2 weeks. Children showed decreased outdoor time (*p* = 0.02–0.008), reduced sunscreen use (*p* = 0.03), reduced rate of reapplication (*p* < 0.001), and unintentional tanning (*p* < 0.001). No changes were reported in UVR exposure, sunburn, or behaviors. Seasonal effects suggested less protection and more tanning in fall versus summer, indicating that reactivity to UVR devices might be considered in prevention research [18].

González Borrego et al. (2025) [19] performed a cross-sectional study in a Spanish community pharmacy (between 2018 and 2023), in which they included 41 adults with skin concerns. Benign lesions were found in 54% of cases and suspicious ones in 46%, most commonly in patients aged over 60, with image-based preliminary diagnoses indicating an accuracy over 85%. The study underscores that pharmacies, in collaboration with dermatologists (and along with progresses in technology), can support early detection and optimization of resources in secondary prevention of skin cancer [19].

In their study, Niu et al. (2022) [20] tested strategies based on digital education for skin self-examination (SSE) in a group of 321 at-risk adults (average age 36.6 years, 56.7% female predominance). Using an online model, they found that high interactivity with customized imagery increased the accuracy of identifying abnormal skin lesions, while increased interactivity with customization or real-person imagery expanded SSE intention over 3 months. Their findings suggest that customization and interactivity optimize digital programs for both melanoma detection and prevention [20].

Mansfield et al. (2024) [21] assessed the multi-institutional SPOTS (Sun Protection Outreach Teaching by Students) program in two cohorts: 1508 adolescents with pre-program and 969 with post-program surveys across the U.S. After one month, significant changes were observed in terms of knowledge, behaviors and attitudes: knowledge of using sunscreen SPF increased by 34.3%, concern that tanning is unhealthy increased by 107.5%, and intention to use sunscreen increased by 27.1% (all *p* < 0.001). Even more, 57.6% reported a wish to increase sunscreen use. These improvements were consistent across skin types and regions, confirming the program’s efficiency in optimizing sun-safe behaviors among adolescents [21].

Hay et al. (2024) [22] analyzed the data derived from a randomized trial based on 593 primary care patients in New Mexico who were offered MC1R genetic testing for assessing skin cancer risk. Fatalism was associated with lower perceived control over risk behaviors, with a few demographic factors such as ethnicity, health literacy, and education (*p* < 0.05), but was not linked in a consistent manner to health beliefs or perception of risk. This study indicates that addressing cancer fatalism is vital for an effective integration of genetic testing in diverse populations [22].

### 4.3. Limitations of the Current Study

This study has several limitations that must be acknowledged together with the interpretation of the results.

To begin with, it was conducted in a single tertiary oncology center, which might limit the reproductivity of the findings and the extension to broader populations, particularly in rural or underprivileged regions of Romania where healthcare-seeking behaviors may differ. Nonetheless, this referral setting also reflects the actual clinical pathway in Romania, where patients with suspicious or advanced lesions are routinely centralized to oncology institutes. For this reason, while our cohort may not capture the full spectrum of rural populations, it remains highly relevant for understanding the profile of patients who reach specialized oncological care.

Additionally, while larger cohorts may always provide higher statistical power, we emphasize that our prospective design (requiring consecutive enrollment of patients with histologically confirmed skin cancer and written informed consent) resulted in a robust dataset of 76 cases. Each patient was documented with complete demographic, clinical, surgical, and psychosocial information, including standardized questionnaires. Collecting such a comprehensive dataset in a prospective manner is challenging, and we believe that the sample size is adequate to provide meaningful preliminary insights.

Furthermore, the evaluation of patients’ understanding levels relied on physicians and is subjective, which could introduce observer bias (despite being structured). Nevertheless, this assessment was grounded in the physicians’ interaction with patients throughout the clinical consultation, which offered a broader perspective on the patient’s level of knowledge. Future research should incorporate validated health literacy instruments to provide a more objective measurement and reduce the subjectivity inherent to physician-based scoring. Also, the study did not include follow-up of patients on longer periods, which would have allowed for a better evaluation of how diagnostic delay and educational level impact survival and clinical outcomes.

Another limitation is caused by not extensively evaluating health literacy, socioeconomic status and cultural factors (only educational level and the place of residence). In addition, self-reported data such as previous participation in awareness campaigns on skin cancer or other sources of information can be subject to recall bias.

It should also be noted that the interval between symptom onset and diagnosis was not based solely on patient recollection; the reported dates were consistently cross-checked against electronic medical records and referral notes. Moreover, patients were usually able to recall major changes such as rapid growth or bleeding with good accuracy. Therefore, while recall bias cannot be completely excluded, its influence in our cohort is likely limited.

Moreover, we did not assess molecular or genetic characteristics of the tumors, which limits the ability to link social determinants with biological aggressiveness. Future research should aim to integrate both molecular profiling and public health variables to better understand how these dimensions interact in shaping outcomes for patients with skin cancer.

### 4.4. Strengths of the Current Study

Our study also has a few strengths that support its findings. First, it was designed in a prospective manner, with consecutive enrollment of all patients, which decreases selection bias and allows for a representative overview of the skin cancer cases that were treated in a tertiary oncology center. Second, the process of data collection was standardized and structured, including a large spectrum of demographic, clinical, pathological, and even psychosocial variables, ensuring an extensive analysis of factors influencing diagnostic delay. Third, our study explored not only the traditional clinical outcomes, but also the patients’ capacity of understanding of their disease, education level, and access to information sources, features that are often missed in oncological research (despite being highly relevant for defining public health interventions).

An additional strength is the systematic use of non-parametric statistical methods that are appropriate for the sample size and distribution of variables, which optimizes the quality of the analyses. Moreover, the inclusion of detailed descriptive statistics and transparency in reporting of medians, interquartile ranges, and *p*-values supports the study’s reproducibility and clarity. In the end, by focusing on a Romanian subgroup, this study provides valuable regional data that might be a start for local awareness campaigns and educational programs, to improve early detection of skin cancer.

## 5. Conclusions

All in all, this prospective study, which was conducted into a tertiary oncology center in Romania, emphasizes the impact of educational level in the field of skin cancer diagnosis. Even though overall diagnostic delays were short, we observed that patients with a higher level of education presented significantly earlier, underscoring the crucial role of health literacy and awareness in defining everyone’s behavior. Geographic residence (urban or rural), together with self-reported preventive practices had a weaker or even non-significant impact, whereas patients’ previous participation in awareness campaigns was linked to a better understanding of their disease.

All these findings show that improvements in public health education combined with sustaining awareness initiatives may be more efficient in promoting early diagnosis compared to focusing extensively on geographic disparities. For populations with poor educational backgrounds or limited health literacy, specific interventions might reduce diagnostic delays and, lastly, improve patient outcomes. Further research performed on larger and more diverse cohorts extended the period of follow-up, and the assessment of socioeconomic factors is mandatory to validate our results and to guide targeted prevention strategies.

## Figures and Tables

**Table 1:** Baseline demographic characteristics of the study population.

Variable	n (%) or Mean ± SD (Range)
Patients	76
Age (years)	58.3 ± 14.5 (26–83)
Sex	Male 40 (52.6), Female 36 (47.4)
Residence	Urban 64 (84.2), Rural 12 (15.8)
Education	University 28 (36.8), Postgraduate 24 (31.6), Post-secondary 12 (15.8), High school 12 (15.8)

**Table 2:** Clinical signs at presentation.

Clinical Sign	n (%)
Rapid growth	56 (73.7)
Bleeding	20 (26.3)
Pain	8 (10.5)
Itching	4 (5.3)
Ulceration	4 (5.3)

**Table 3:** Physician-assessed patient understanding.

Score	n (%)
1—No understanding	2 (2.6)
2—Limited	8 (10.5)
3—Moderate	20 (26.3)
4—Good	28 (36.8)
5—Full	18 (23.7)

**Table 4:** Summary of inferential statistical results.

Hypothesis Tested	Statistical Test	Result (*p*-Value)	Interpretation
Education vs. time to diagnosis	Kruskal–Wallis	*p* = 0.043	Significant difference across education groups
Urban vs. rural time to diagnosis	Mann–Whitney U	*p* = 0.483	Not significant
Understanding vs. diagnostic delay	Spearman correlation	rho = −0.17, *p* = 0.15	Not significant
Self-examination vs. delay (short/long)	Chi-square	*p* = 0.86	Not significant
Awareness campaign vs. understanding	Chi-square	*p* < 0.001	Significant association

**Table 5:** Diagnostic interval by educational level.

Educational Level	n	Median (Weeks)	IQR (Weeks)
High school	12	3.0	2.0–4.0
Post-secondary	12	3.0	2.0–4.0
University	28	3.0	3.0–6.0
Postgraduate	24	2.5	2.0–3.0

**Table 6:** Diagnostic interval by residence.

Residence	n	Median (Weeks)	IQR (Weeks)
Urban	64	3.0	2.75–4.0
Rural	12	2.0	2.0–12.0

## Data Availability

Data are available upon reasonable request from the corresponding author.
